# A Retrospective Study on the Factors Associated with Long-Stay Hospitalization in a Child Neuropsychiatry Unit

**DOI:** 10.3390/healthcare9091241

**Published:** 2021-09-21

**Authors:** Silvia Zanato, Marina Miscioscia, Annalisa Traverso, Miriam Gatto, Mikael Poli, Alessia Raffagnato, Michela Gatta

**Affiliations:** 1 Child and Adolescent Neuropsychiatric Unit, Department of Women’s and Children’s Health, University Hospital of Padua, 35128 Padua, Italy; silvia.zanato_01@aopd.veneto.it (S.Z.); annalisa.traverso@aopd.veneto.it (A.T.); alessia.raffagnato@aopd.veneto.it (A.R.); michela.gatta@unipd.it (M.G.); 2 Department of Developmental Psychology and Socialization, University of Padua, 35131 Padua, Italy; Miriam.gatto@studenti.unipd.it (M.G.); Mikael.poli@studenti.unipd.it (M.P.)

**Keywords:** mental health disorders, psychopathology, pediatric psychiatric hospital, inpatients, long-stay hospitalization, children and adolescents

## Abstract

The past twenty years have seen a rapid increase in acute psychiatric symptoms in children and adolescents, with a subsequent rise in the number of psychiatric hospitalizations. This paper aims to: (a) describe the epidemiology of hospitalizations and some of the clinical and sociodemographic characteristics of pediatric patients admitted to a regional referral Complex Operative Child Neuropsychiatry Hospital Unit in Northeast Italy and (b) identify potential factors correlated with the length of hospital stay. Methods: 318 (M = 12.8 years; SD = 3.11; 72% Female) patients hospitalized for mental health disorders from 2013 to 2019. Results: Around 60% of hospital admissions occurred via the emergency room, mostly due to suicidal ideation and/or suicide attempts (24%). Affective disorders were the most frequent discharge diagnosis (40%). As for factors correlated with length of hospital stay, we found significant links with chronological age, way of hospital admission, cause of admission, discharge diagnosis, presence of psychiatric comorbidity, family conflict, and psychiatric family history. Conclusions: These results provide information about global characteristics associated with the length of psychiatric hospital stays in pediatric patients and provide a basis on which specific precautions can be hypothesized with the aim of developing more focused treatments.

## 1. Introduction

Mental health disorders affect around 15–20% of the global pediatric population, a percentage that was destined to increase [1,2] even before the COVID-19 pandemic, which has led to more adverse mental health consequences [3]. In the Global Burden of Disease (GBD), the World Health Organization (WHO) reports how psychopathology constitutes one of the five most common causes of morbidity, disability, and mortality in childhood and adolescence [4,5]. Throughout 2016, mental illness and substance use disorders contributed to lower quality of life across all countries, regardless of socioeconomic status.

However, the treatment rates for such conditions remain low. In high-income countries, the prevalence of the most common disorders has not changed despite the increase in treatment coverage [6]. More specifically, depressive disorders represent the third most common cause of psychological distress in the child population, with a four times higher percentage for adolescents than younger children [6]. Furthermore, suicide constitutes the third leading cause of death among adolescents [6]. In this age group, acute psychiatric conditions are the main cause of admission to mental healthcare services [2,7,8,9,10,11].

In Italy, the number of pediatric-age patients accessing the emergency department due to acute psychiatric symptoms has almost doubled in the past 15 years; recent studies describe self-injury and aggressive behaviors as the most common reasons for admission [12].

Simultaneously, the number of pediatric psychiatric hospitalizations, which are essential whenever the worsening of symptoms is not manageable in other health care contexts and whenever the patient shows behaviors that can constitute a danger to him/herself and others, is rising [13,14].

In the literature, little information emerges regarding hospitalization trends [13]. Nonetheless, the demographic and clinical factors most associated with psychiatric hospitalizations during child development appear to be adolescence, suicidal ideation and/or suicide attempts (more frequent among females), psychomotor agitation (more frequent among males), a psychiatric family history, past hospitalizations, psychiatric comorbidity, a history of victimization (bullying, physical, psychological, and/or sexual abuse, neglect, and witnessing violence), and academic and relational difficulties [2,7,14,15,16,17,18]. In particular, a large number of studies identify parental psychiatric illness as one of the most significant risk factors for pediatric-age psychiatric hospitalization [16,19,20,21].

Undoubtedly, an additional factor that ultimately influences hospitalization is the availability of hospital beds and the presence of specific wards and services for mental disorders during development [22,23]. In this regard, the international scene is characterized by inhomogeneous health policies and considerable organizational differences in pediatric psychiatric services’ management [13,24].

The Italian situation does not appear different, and in some cases, it proves to be even more serious due to the significant difficulties related to the scarcity of services and dedicated structures and the lack of timeliness in identifying disorders and carrying out interventions [10]. More specifically, only 30% of hospitalizations are carried out in a Child Neuropsychiatry ward, while the remaining 70% are distributed in other pediatric wards not specifically dedicated to such pathologies, or in adult Psychiatric Diagnosis and Care Services [12,13].

In this context of limited resources, the Child Neuropsychiatry Unit of the Hospital-University of Padua–Department of Women’s and Children’s Health–is in charge of hospital admission and care for children and adolescents aged 0–17 with chronic or acute psychiatric disorders. Each hospitalization is managed by a multidisciplinary team, which involves different therapeutic approaches (pharmacological, psychotherapeutic, and relational–educational) aimed at supporting, containing, and caring for the patient and his/her caregivers.

The length of stay is associated with an increased risk of hospital readmission and factors such as greater symptom severity, specific clinical diagnoses (e.g., psychosis and mood disorders), self-injury and suicidal behaviors, and poor family functioning [25]. The discussion around length of stay is pressing in light of the increase in access requests and in the face of the scarcity of child neuropsychiatry hospital beds, particularly for emergency psychiatric hospitalizations. Appropriate bed turnover rates are necessary in order to meet incoming access requests and minimize pediatric psychiatric patients’ admissions to adult care facilities.

## 2. Materials and Methods

### 2.1. Aims

The study aims to examine the characteristics of patients admitted from 1 January 2013 to 31 May 2019 (total duration six years and five months) to a regional referral Complex Operative Child Neuropsychiatry Hospital Unit (UOC-NPI) dedicated to psychiatric hospitalizations in both emergency and nonemergency conditions within a pediatric hospital in Northeast Italy.

In particular, the aims of our study are: (a) to evaluate the annual frequency of hospitalizations during the reference period and to analyze the sociodemographic and clinical characteristics of patients (features identified by clinical interviews, questionnaires, and other tools, diagnostic assessment, and discharge diagnosis); (b) to evaluate the length of hospital stay and identify the sociodemographic and clinical factors related to it.

### 2.2. Participants

The study included all patients aged ≤ 17 years admitted to the NPI service for at least two days from 1 January 2013 to 31 May 2019. The total was 318 subjects with a mean age of 12.8 years (SD = 3.11; range 2–17 years). Female patients were prevalent: 230 females (72%) versus 88 males (28%). A total of 91% percent of the subjects were Caucasian and 9% non-Caucasian (3% Latin American; 3% African; 2.5% Asian; 0.5% other ethnicity). Eighty-one percent were born in Italy and 19% had immigrated to Italy.

In the child and adolescent population analyzed, 85.5% were born at term and 11% preterm (10.1% Low Gestational Age; 0.9% Very Low Gestational Age); these data were not available for 3.5% of subjects. Furthermore, 44.3% of our sample suffered from a chronic physical pathology (such as headache, epilepsy, metabolic disorder, or genetic syndrome) concomitant with a psychiatric pathology.

### 2.3. Procedure

Within the Complex Child and Adolescent Neuropsychiatry Hospital Unit (UOC-NPI) at a Pediatric Hospital Clinic, patient management is the responsibility of an interdisciplinary team consisting of doctors, psychologists, educators, nurses, and public health social workers. The multidisciplinary evaluation carried out during hospitalization includes neuropsychiatric interviews for the patient and parents; neuropsychological assessment to evaluate the patient’s cognitive and executive functioning; administration of projective tests and structured and/or semi-structured questionnaires; and the observation of family interactions through the Lausanne Trilogue Play (LTP) procedure [26]. This complete multidisciplinary evaluation takes at least 10 days. Clinicians are divided into small multidisciplinary patient-tailored teams. Each of these teams (often composed of four or more clinicians: a neuropsychiatrist supervisor, two resident neuropsychiatrists, and a psychologist) are dedicated to patient management (medical examinations, contacts with local mental health services, diagnostic and therapeutic interviews with the patient (at least three/week), clinical interviews with the parents (at least one/week), observation of family interactions, neuropsychological and clinical assessment, clinical discussion, and definition of the daily care plan).

During hospitalization, individual and group educational–rehabilitative interventions are carried out. The team also participates in multiple scheduled meetings to discuss diagnostic–therapeutic projects and treatment plans for all patients (biweekly team meetings), monthly supervisions with external experts, and monthly meetings between the hospital and local Child Mental Health services. The reference manual for the psychiatric diagnosis of children and adolescents consists of Chapter V (Mental and Behavioural Disorders) of the 10th revision of the International Statistical Classification of Diseases and Related Health Problems (ICD-10) by the World Health Organization (1994) [27]. For clinical and research practice, the DSM 5 (Diagnostic and Statistical Manual of Mental Disorders, APA 2013) is also used [28].

The present study was conducted through the review of medical records and the consultation of telematic archives. The data were extrapolated from the discharge letters and from the reports of clinical interviews carried out with patients and family members. Sociodemographic and clinical information relating to the patients were collected from medical charts (way of hospital admission, reason for admission, posthospital treatment indication, discharge diagnosis, psychiatric comorbidity, and scores obtained from the following standardized questionnaires: Child Behaviour Checklist, Children’s Depression Inventory and Multidimensional Anxiety Scale for Children). Information relating to the family unit was also collected.

### 2.4. Tools

CBCL and YSR Achenbach questionnaires [29,30] are among the most commonly used scales for rating child and adolescent behavior, used internationally in the clinical setting and in research. We used self-completed questionnaires: one completed by the adolescents (Youth Self-Report 11-18) and one completed at least by one parent or both (Child Behaviour Checklist 1.5-5 and 6-18 version). These questionnaires yielded two profiles: one for competences (activities, social functioning, and school performance), which provided information about the adolescent’s level of personal autonomy and social skills and about how well the adolescent performs in sports, hobbies, and school; the other for behavioral and emotional problems, both of which can be assessed as “normal”, “borderline”, or “clinical” on eight specific syndrome scales. The syndrome scales relating to the various possible clinical pictures are aggressive behavior, anxiety/depression, attention problems, rule-breaking behavior, somatic complaints, social problems, thought problems, and withdrawal. The problems are grouped into internalizing problems (anxiety, depression, withdrawal, and somatization), externalizing problems (aggressive and rule-breaking behavior), and other problems (social problems, thought problems, and attention problems).

The Children’s Depression Inventory (CDI) is composed of 27 items assessing feelings, behavior, and thoughts associated with depression in childhood and adolescence aged 7–17. Respondents are asked to choose one of three sentences that best describe their feelings in the previous two weeks. Each answer is then assigned a score ranging from 1 to 3: the total score is obtained from the sum of the single scores (19 represents the cutoff above which the presence of depressive traits can be determined) [31]. In this study, we employed the Italian version of the CDI.

The Multidimensional Anxiety Scale for Children (MASC) evaluates anxiety symptoms in children and adolescents aged 8–19. It is a self-reporting questionnaire consisting of 39 items to which the subject is asked to respond using a Likert scale (never/rarely/sometimes/always). The final profile provides information related to four main factors, three of which can be divided into two subfactors. The main factors are physical symptoms, harm avoidance, social anxiety, and separation anxiety [32].

### 2.5. Statistical Analysis

Data analysis was carried out using the JAMOVI statistical software package [33]. In order to control, explore, and describe the quantitative and qualitative variables taken into consideration, we carried out a descriptive analysis. Therefore, total and cumulative percentage frequency indices and measures of central tendency (particularly, the mean) and variability (particularly, the standard deviation and coefficient of variation) were obtained. In addition, we decided to calculate the correlation coefficient with the aim of observing any potential relationships between the sociodemographic and clinical variables, the scores on the psychodiagnostics tests (CBCL, YSR, MASC, and CDI), and the length of hospitalization. Due to the strongly asymmetrical distribution of the “length of hospitalization” variable, we decided to calculate Spearman’s rank correlation coefficient (rho).

In addition, we applied the Kruskal–Wallis nonparametric test in order to identify whether there were statistically significant differences between the way of hospital admission, the reason for hospitalization, the primary discharge diagnosis, and the length of stay. In order to identify statically significant differences between the T scores relating to “internalizing problems” and “externalizing problems” areas (contained in the CBCL and in the YSR) and the patients’ sex, we applied Mann–Whitney’s nonparametric U test. Finally, the Chi-Square (χ^2^) test was applied to investigate any potentially significant effect of sex on the reason for hospital admission and on the primary diagnosis.

In order to analyze specific predictors for the length of hospitalization, we ran a logistic regression. For this purpose, we considered, based on our specific modality of multidisciplinary evaluation, a ‘brief’ length of stay to be up to 12 days, and we considered more than 12 days a ‘long’ hospitalization.

## 3. Results

**Figure 1:**
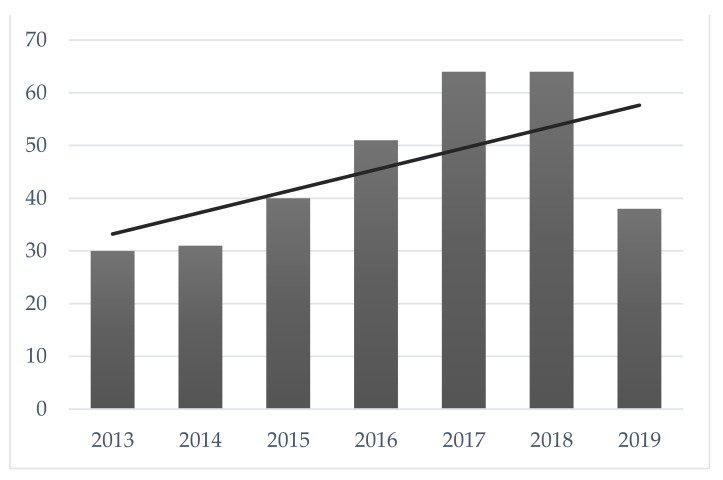
Distribution of the number of hospitalizations (January 2013–May 2019)).

Over the years, we have observed an increase in the number of hospitalizations (Figure 1). A growing trend has been registered particularly from 2017, the year in which the service was established as a Complex Operative Unit (UOC), thus determining greater autonomy and organization within the Pediatric Department, also in terms of greater availability of clinical staff.

The percentage frequencies related to the way of hospital admission show that 59.7% took place via the emergency department, 17.9% via transfer from other hospitals or services, 13.8% via scheduled hospitalization, 5.7% via an outpatient clinic, and 2.8% through counseling. Suicidal ideation and suicide attempt (24.2%) were the most frequent causes of hospital admission, followed by psychomotor agitation (17.3%). Other causes were eating difficulties and mismanagement (13.8%), functional symptoms (9.1%), non-suicidal self-injury (8.5%), anxiety symptoms (7.9%), acute psychotic symptoms (3.8%), and other (such as diagnostic assessment in metabolic or genetic disorders or psychopharmacological assessment) (15.4%).

At discharge, the largest number of patients (39.9%) received a diagnosis of affective disorders (ICD-10: F30–F39), followed by phobic, stress-related, and somatic symptom syndromes in 17.6% of patients (F40–F48), and behavioral disturbances in 12.3% (F90–F98) (Table 1). Furthermore, the majority of patients showed at least one comorbidity with other mental health disorders (one comorbidity in 51.6% of patients, more than one comorbidity in 17.9% of cases). Upon discharge, most patients (52.8%) were referred to level I child neuropsychiatry local services, 33.9% were referred to level II territorial structures (which offer both outpatient and semi-residential services), 4.7% to regional eating disorder treatment centers, 3.5% were admitted to a residential youth care center (mainly educational and rehabilitation centers or therapeutic centers), and 5% were transferred to other wards (e.g., adult psychiatric diagnosis and care services) or to other hospital/rehabilitation facilities.

Data obtained from structured questionnaires were collected and analyzed: 148 mothers and 88 fathers completed the CBCL (for 83 of the global sample we had both CBCL completed by both parents), thus providing information on the emotional–behavioral profile of their children. Table 2 presents the mean and standard deviation of the CBCL score and the percentage, among subjects, of those who passed the clinical cutoff. Furthermore, from the patient self-report questionnaires, it emerged that 163 subjects had completed the YSR, 128 the MASC, and 68 the CDI (Table 2).

### Factors Associated with Length of Stay

The variable of interest “length of hospital stay” has a strongly skewed distribution (M = 23.8; SD = 21; Min = 2; Max = 116; K = 2.35; A = 1.58; Shapiro–Wilk *p* < 0.001). The statistical analyses carried out on the variables relating to the patient yielded a positive correlation between chronological age and length of hospitalization (rho = 0.129, *p* = 0.022)—that is, as the patient’s age increased, the number of inpatient days also increased. With reference to psychiatric comorbidity, we observed a positive correlation between comorbidity with another psychiatric disorder and length of hospital stay (rho = 0.194, *p* ≤ 0.001), and comorbidity with two or more psychiatric disorders and length of hospital stay (rho = 0.152, *p* = 0.007). This underlined that as the number of psychiatric comorbidities increased, the duration of hospitalization also tended to increase. As for the remaining variables, we did not find any statistically significant correlation between length of hospital stay and sex (rho = −0.041, *p* = 0.462), ethnicity (rho = −0.073, *p* = 0.196), immigration (rho = −0.002, *p* = 0.976), number of previous hospitalizations (rho = 0.039, *p* = 0.491), chronic disease (rho = 0.016, *p* = 0.774), and preterm birth (rho = 0.028, *p* = 0.631).

The way of hospital admission had a significant impact on length of stay: χ^2^ (3) = 17.2, *p* < 0.001. In a paired comparison test, we found that the number of inpatient days was greater for subjects who were admitted via the Emergency Department (M = 26.4 days), and it was shorter for subjects who were admitted following scheduled hospitalization (M = 13.5 days).

The main diagnosis also played a significant role in the duration of hospital stay (χ^2^ (8) = 50.9, *p* < 0.001): we found a statistically significant difference between affective disorders (ICD-10: F30–F39) and anxiety disorders (ICD-10: F40–F48), and between anxiety disorders and eating disorders (ICD-10: F50): specifically, the length of stay was longer for eating disorders (M = 36.1 days) and psychosis and affective disorders (M = 24.6 days) than it was for anxiety disorders (M = 16.2 days). The length of hospital stay was positively correlated with intrafamily conflict (parental conflict and/or parent–child conflict) (rho = 0.168, *p* = 0.003) and with a family history of psychiatric disorders (rho = 0.169, *p* = 0.003).

Moreover, we found statistically significant correlations between the scores on the self-report questionnaires and behavior-rating scales and the variable of interest “length of hospital stay”. More specifically, we found links between length of hospital stay and some of the caregiver-reported CBCL categories (internalizing problems, affective disorders, post-traumatic symptoms, and social withdrawal) and the patient’s answers to the YSR questionnaire (Table 3). Positive correlations also emerged between length of hospital stay and CDI scores (which rate the severity of depressive symptoms in children and adolescents) (N = 68) on the following scales: total score (rho = 0.413, *p* ≤ 0.001), negative mood (rho = 0.282, *p* = 0.020), and negative self-esteem (rho = 0.330, *p* = 0.006). Finally, we found positive correlations between the MASC self-report questionnaire (N = 128), which assesses anxiety symptoms in children and adolescents, and the length of stay, in the following MASC areas: social anxiety (rho = 0.238, *p* = 0.007) and anxiety disorder index (ADI) (rho = 0.229, *p* = 0.009) (Table 4).

We observed a prevalence of female inpatients for all causes of hospital admission, with the exception of psychomotor agitation and functional symptoms, which were more frequent in males (χ^2^ (7) = 53.2, *p* < 0.001) (Table 5). Furthermore, there appeared to be a statistically significant difference between sex and discharge diagnosis (χ^2^ (8) = 33.6, *p* < 0.001), with a clear difference in the M:F ratio for affective disorders and eating disorders (more frequent in females) and behavioral disturbances (more frequent in males) (Table 5).

The results of logistic regression (Table 6) analyses to assess the predictors of length of hospitalization with clinical and sociodemographic variables indicated that there was a significant association between age, family history of psychiatric disorders, cause of admission (non-suicidal self-injury, psychomotor agitation/aggressive behavior, eating difficulties and mismanagement, and psychotic symptoms) and hospital admission (χ^2^ (14) = 58.2, *p* < 0.001; R^2^_N_ = 0.230).

## 4. Discussion

The main objective of this study was to evaluate the frequency and duration of hospitalizations that occurred at a regional referral Complex Operative Child Neuropsychiatry Hospital Unit in Northeast Italy between January 2013 and May 2019, analyzing some of the clinical and sociodemographic characteristics of the patients and their parents. This study highlights an increase in psychiatric hospitalizations of children and adolescents during the reference period and is therefore consistent with the findings that have been reported in the existing literature [2,13,34]. On the one hand, the rapid increase in assistance requests directed at Complex Operative Child Neuropsychiatry Hospital Units could be in part due to the greater awareness and knowledge of parents, pediatricians, and teachers regarding neuropsychiatric disorders [35]; on the other, it could reflect an increase in the negative outcomes resulting from the interaction between biological vulnerability, negative experiences, and environmental factors [36].

Similar to what some studies have reported [7,16], the average age of the patients can be traced back to preadolescence. Among hospitalized patients, there is also a clear prevalence of females. With regard to the way of hospital admission, in line with other studies [7,8,37], about 60% of admissions occurred in acute conditions following access to the Emergency Department. The main way of hospital admission is represented by suicidality, in line with national and international data [2,7,8,9]. With respect to sex, about 30% of the female patients in the sample examined arrived at the Child Neuropsychiatry unit due to suicidal ideation or a suicide attempt, versus 10% of the males. This prevalence also emerges in the literature–in fact, girls are more likely to experience suicidal ideation and/or attempt suicide than boys [38,39]. Affective disorders are the main discharge diagnosis, representing about 40% of the sample. In this sense, the association between mood disorders and suicidality is well reported in the literature [40,41,42].

Furthermore, there appears to be a significant impact of sex on the main diagnosis: on the one hand, more females receive a diagnosis of affective disorder than males. On the other, 24% of males receive a diagnosis of behavioral disorder compared to 8% of females. These percentages further confirm the results of previous studies, according to which internalizing disorders are more prevalent in females and, vice versa, externalizing disorders are more prevalent in males [8,43,44], as gender differences are linked to several different factors: genetic vulnerability, hormonal and neuropsychological changes, differences in personality, different coping styles, and cultural and social beliefs [43,45,46].

Upon discharge, most of the subjects were referred to local NPI services located in different parts of the Region for pharmacological monitoring, psychotherapeutic, and/or semi-residential treatment. This information highlights the importance of collaboration between healthcare and social and local mental health services in the field of developmental neuropsychiatry in order to ensure continuity throughout the different phases of treatment [35]. In this regard, it is necessary to facilitate the transition process from hospital to local mental health service as much as possible, as an uncoordinated territorial transfer could cause a new emergency hospital admission and subsequent hospitalization [10].

In the literature, there are many studies aimed at identifying the variables related to psychopathology and psychiatric hospitalization [47,48,49], in our study we specifically investigated various factors related to the duration of psychiatric hospitalizations during child development. For this reason, we analyzed possible correlations with the length of stay. First, it is important to specify that the average length of hospitalization (M = 23.8 days) clearly exceeds that reported by some studies in the literature (about seven days) [13,14]; various studies have shown that this datum is influenced not only by the clinical characteristics of the patient but also by the organizational structure of the regional health and social care services’ network [50,51], and this is clear in our local organization. The patient is discharged after the acute clinical picture has stabilized, after the patient’s family has agreed to comply with the treatment project, and the treatment plan has been developed and shared with the territorial child neuropsychiatry services’ contact persons. The length of hospital stay can therefore be linked both to the clinical severity of the cases and to the time it takes to formulate adequate supported discharge projects that can guarantee the prompt care of the patient after discharge. Sharing the patient and family treatment project could help to prevent readmission to hospital.

With respect to personal information, we found that age correlates positively with the length of hospital stay, which is longer for adolescents compared to children. It is easy to link these data with a greater complexity of the clinical pictures linked to the difficulties that adolescence entails (i.e., psycho–physical, identity, and affective–relational changes). In this regard, we find it necessary to highlight the increasing issue of psychiatric emergencies during adolescence, which has characterized the clinical and welfare challenges of recent years [52,53,54,55,56].

In relation to sex, although there is a clear prevalence of hospitalizations for female subjects compared to males, we found no significant correlation between this variable and length of hospital stay.

In contrast with what some international studies have highlighted [57,58], ethnicity and immigration were not significantly associated with the length of hospital stay. In their study, Gattoni and colleagues (2015) [59] observed lower rates of health services utilization among immigrants, an aspect probably associated mainly with linguistic and cultural factors. Overall, studies show that linguistic and cultural factors are often associated with longer first appointment access times, less frequent clinical examinations, and greater difficulty understanding doctors’ explanations, and can therefore lead to lower levels of satisfaction and health service utilization [60]. In the present study, prematurity and the presence of a chronic medical condition, which often constitute predisposing factors to psychopathology [14,57,61,62], were not significantly associated with the duration of hospitalization. The present research is one of few studies, to the authors’ knowledge, that investigated the predictors of length of hospitalization on the Italian child neuropsychiatric population. The results highlight that age, family history of psychiatric disorders, and mode and causes of hospital admission predict a longer stay hospitalization. The mode of hospital admission has a significant impact on the length of hospitalization. As expected, the duration of hospitalization tends to be longer for those who are admitted via the Emergency Department, with a duration of just under a month. As a matter of fact, these cases are more serious and require a longer period for clinical stabilization, as studies on access to pediatric emergency departments report [8,9]; more complex cases also often require longer times for the definition of a post discharge care plan: because they involve a multidisciplinary team, such treatment plans require the collaboration of a greater number of health services and more organizational steps. 

In particular, between the causes of admission, non-suicidal self-injury, psychomotor agitation/aggressive behavior, eating difficulties and mismanagement, and psychotic symptoms play a significant role in determining the length of hospital stay. In line with the literature, eating disorders require longer hospitalizations [14], likely due to some of the features of these conditions, such as ambivalence towards treatment, psychiatric comorbidity (particularly with depression and suicidality), and organic sequelae which require slow and gradual medical stabilization [63]. Previous research findings also support the relationship between psychotic conditions and increased length of stay [64]. Finally, multiple comorbidities at discharge are also positively correlated with the length of hospitalization, underlining how a greater complexity of the clinical picture and higher psychiatric comorbidity correspond to an increased length of hospital stay [65]. Logistic regression does not confirm this aspect as a predictor of long-stay hospitalization.

With regard to family characteristics, the length of hospitalization is positively correlated with intrafamily issues, understood as parental conflict and/or conflict between parents and children: as levels of intrafamily conflict increase, the length of hospitalization also increases. The family environment is known as a protective factor, rather than a risk one, for the development of psychopathology. As a risk factor, parental conflict, rather than parental separation alone, is the aspect that most affects both the potential development of psychopathology and its severity in children [66]. Other elements that have been identified as risk factors for the emergence of psychopathology are inadequate emotional exchanges, lack of support, and low cohesion and poor communication in the family system [66,67,68,69].

While the aforementioned correlation highlights the role of environmental factors on the development of psychopathology, the positive correlation between a family history of psychiatric disorders and the length of hospitalization (a greater family history of psychiatric illness is linked to longer hospitalizations) underlines the influence that genetic vulnerability plays parallel to the role of experience. In fact, a strong association has been observed in the literature between a family history of psychiatric disorders, particularly when the parents are affected, and the risk of psychopathology in children [16,70,71], with a threefold higher risk for the child of being hospitalized with one or more psychiatric conditions [16]. In addition, a problematic familiar context could bring about a more difficult stabilization of the acute phase: in our unit, the presence of a parent is requested to support the patients during hospitalization, so at the relational level, family psychopathology could compromise parents’ caregiving abilities and the quality parent–child interactions [70]. 

While all family psychopathologies have been identified as risk factors, affective disorders, in particular, can affect child development through parent–child role reversal: the child could take on the responsibility of caring for the sick parent—a function that is in stark contrast with the child’s typical needs during this phase of life [70]. With respect to tests and questionnaires, from the analysis of parent’s CBCL reports we found a discrepancy between the scores provided by the mothers and those provided by the fathers. Mothers’ ratings generally appear higher and there is greater correlation between CBCL areas that fall in the “clinical” range and length of hospitalization. The results show that the length of stay increases with higher levels of anxiety–depression problems, withdrawal, somatic complaints, thought problems, internalizing problems, externalizing problems, total problems, affective disorders, attention disorders, obsessive compulsive symptoms, and post-traumatic stress symptoms. The longer duration of hospitalization is also inversely associated with the child’s social skills.

The analysis of fathers’ CBCL ratings yielded a single positive correlation: the increase in the length of hospitalization is associated with greater thought problems.

The analyses of patients’ self-report questionnaires (YSR 11-18) show an association between length of stay and greater social, attentional, anxiety–depression, withdrawal–depression, and internalizing problems, and obsessive compulsive and post-traumatic symptoms. Furthermore, in line with the ratings provided by both parents, the length of hospitalization is positively correlated with thought problems.

A different perception of skills, emotional, and behavioral issues as reported in behavior-rating scales by different subjects (mother, father, and patient) is highlighted both in the present study and in national and international literature [72,73,74,75]. The different perception of psychological–psychiatric difficulties by different family members could be due to the stigma that is still attached to such pathologies, to potential feelings of guilt, to misinformation, and/or to the trivialization of psychological distress in children [7].

An essential element of the therapeutic process during child development, in addition to appropriate care of patients and families, is the collaboration between child neuropsychiatry services and other health, social, and educational services in order to guarantee continuity in the different phases of treatment [35]. In this regard, it is necessary that the transition process between services be facilitated as much as possible [10] and this leads to an increase in the total number of inpatient days. In order to reduce the length of stay, as well as to facilitate the referral process and ensure prompt territorial care, hospital–territory collaborations represent a fundamental element in the management of child neuropsychiatric hospitalizations. 

Considering the factors significantly correlated with the length of hospitalization that have been highlighted in our study, it is fundamental to promptly activate intra- and extrahospital programs aimed at planning the psychodiagnostic and therapeutic steps necessary for the diagnosis and clinical stabilization of the patient, thorough assessment of the child’s family context, and the connection with extrahospital services for the definition of assisted discharge programs. As the length of stay correlates with negative outcomes such as readmission, policy strategies are desirable to improve the discharge systems, enhance hospital and peripheral services, and promote mental health wellbeing. 

## 5. Conclusions

These results provide some clinical and sociodemographic characteristics associated with the length of stay of psychiatric hospitalizations of pediatric patients. Moreover, our study highlights an increase in psychiatric hospitalizations of children and adolescents in recent years reflecting an increase in vulnerabilities in pediatric patients. The present study is not exempt from limitations, first of all linked to the incompleteness of some sociodemographic and clinical information, as it was retrieved retrospectively without a longitudinal design. The inhomogeneity of the age range in the tools used and their incomplete compilation for all subjects or all parents represent another limitation of this study. It is important to underline that the magnitude of correlations observed is, in some cases, very low; further research is needed to understand whether this aspect represents a specific limitation of our study or a feature of the relationship between the variables studied. In fact, in a clinical naturalistic study, the population observed is very heterogeneous; naturalistic research needs a precise methodology and good familiar compliance, which is often a difficult goal to achieve in the acute phase.

However, taking these limitations into account, the study proves to be innovative, as it focuses on various factors associated with the duration of psychiatric hospitalizations during child development, a topic that is not often dealt with in the literature but is important to investigate due to the increasing rate of child and adolescent psychiatric inpatients. In this regard, it would be beneficial to conduct further studies using a multicenter research methodology for analyzing hospitalization trends within the Italian context.

## Figures and Tables

**Table 1:** Total percentage frequency of discharge diagnosis (ICD-10).

ICD-10 CODE	Diagnosis	Percentage
F30–F39	Mood (affective) disorders	39.9%
F40–F48	Anxiety and obsessive compulsive disorders	17.6%
F90–F98	Behavioral disorders	12.3%
F50	Eating disorders	10.4%
F44–F45	Somatoform and conversion disorders	6%
F20–F29	Psychotic disorders	4.4%
F80–F89	Disorders of psychological development	3.1%
	Other	3.8%
	No diagnosis	2.5%

**Table 2:** Descriptive statistics (M; SD; Clinical %) for CBCL, YSR, MASC, and CDI scores.

CBCL	Mother (*n* = 148) M(SD)	CV	Clinical (%) MD	Father (*n* = 88) M (SD)	CV	Clinical (%) FD
Anxious/Depressed	69.6 ± (10.6)	15.23%	45.3%	66 (± 9.62)	14.57%	31.8%
Withdrawn/Depressed	70.2 ± (12)	17.09%	43.2%	66.6 (± 9.68)	14.53%	23.9%
Somatic Complaints	65.5 ± (9.02)	13.77%	25.7%	63.3 (± 8.53)	13.47%	19.3%
Social Problems	63.6 ± (8.85)	13.91%	14.9%	60.5 (± 7.68)	12.69%	5.7%
Thought Problems	67.2 ± (8.70)	12.95%	36.5%	64.6 (± 8.15)	12.62%	18.2%
Attention Problems	61.6 ± (9.93)	16.12%	16.2%	59.1 (± 7.69)	13.01%	9.1%
Rule-Breaking Behavior	60.5 ± (8.65)	14.30%	15.8%	59.4 (± 8.86)	14.91%	14.9%
Aggressive Behavior	62.1 ± (9.76)	15.72%	17.8%	60.3 ± (8.03)	13.32%	10.3%
Internalizing Problems	69.1 ± (10.6)	15.34%	76.4%	66.8 ± (8.40)	12.57%	70.5%
Externalizing Problems	59.7 ± (11.4)	19.09%	35.1%	58.9 ± (10.3)	17.49%	33%
Total Problems	66.1 ± (9.15)	13.84%	68.2%	63.2 ± (8.47)	13.40%	53.4%
Affective Problems	71.3 ± (10.2)	14.30%	53.4%	68.5 ± (9.32)	13.60%	37.5%
Anxiety Problems	65.8 ± (7.35)	11.17%	31.1%	64.3 ± (7.26)	11.29%	17%
Somatic Problems	63.2 ± (9.27)	14.67%	13%	61.3 ± (8.58)	13.99%	10.2%
Attention Deficit/Hyperactivity Problems	58.4 ± (7.82)	13.39%	9.5%	57.4 ± (7.33)	13.26%	4.5%
Oppositional Defiant Problems	59.7 ± (8.23)	13.78%	10.8%	59.4 ± (7.88)	13.26%	6.8%
Conduct Problems	60.2 ± (9.09)	15.10%	14.4%	58.7 ± (8.16)	13.90%	10.3%
Sluggish Cognitive Tempo	62 ± (8.45)	13.63%	15%	58.3 ± (6.85)	11.75%	2.3%
Obsessive Compulsive Problem	66.7 ± (11.5)	17.24%	33.3%	63.5 ± (7.83)	12.33%	13.6%
Post-traumatic Stress Problems	70.4 ± (11.8)	16.76%	45.5%	66.9 ± (8.35)	12.48%	27.6%
**YSR**	***n* = 163** **M (±SD)**	**CV**	**C (%)**			
Anxious/Depressed	66.4 ± (12.7)	19.13%	31.9%			
Thought Problems	64.1 ± (10.9)	17.00%	22.1%			
Attention Problems	59.6 ± (9.86)	16.54%	12.3%			
Rule-Breaking Behavior	55.4 ± (6.86)	12.38%	4.4%			
Aggressive Behavior	56.5 ± (6.96)	12.32%	3%			
Internalizing Problems	67 ± (12.6)	18.80%	59.5%			
Externalizing Problems	53.7 ± (10.7)	19.92%	15.3%			
Total Problems	61.8 ± (11.9)	19.25%	44.8%			
Affective Problems	68.6 ± (13.8)	20.12%	43.8%			
Anxiety Problems	61 ± (9.30)	15.24%	17.8%			
Somatic Problems	62 (± 9.56)	15.42%	16%			
Attention Deficit/Hyperactivity Problems	56.5 ± (6.81)	12.05%	2.5%			
Oppositional Defiant Problems	57.3 ± (8.43)	14.71%	11.1%			
Conduct Problems	56 ± (7.61)	13.59%	6.2%			
Obsessive Compulsive Problems	63.2 ± (9.47)	14.89%	21%			
Post-traumatic Stress Problems	65.4 ± (11.7)	17.89%	27.8%			
**CDI**	***n* = 68** **M (±DS)**	**CV**	**C (%)**			
CDI Total	27.2 ± (8.17)	30.04%	85.3%			
Negative Mood	10.1 ± (4.46)	44.16%	79.4%			
Negative Self-esteem	8.37 ± (3.17)	37.87%	80.9%			
Interpersonal Problems	8.37 ± (3.94)	47.07%	57.4%			
**MASC**	***n* = 128** **M (±SD)**	**CV**	**C (%)**			
MASC Total	56.3 ± (11.3)	20.07%	35.2%			
Physical Symptoms	57.2 ± (10.7)	18.71%	35.2%			
Harm Avoidance	43.4 (± 11.2)	25.81%	4%			
Social Anxiety	58 ± (13.2)	22.76%	42.2%			
Separation/Panic	55.1 ± (12.7)	23.05%	28.1%			
Anxiety Disorder Index (ADI)	52.4 ± (13.2)	25.19%	26.6%			

*n* = Sample size; C = Clinical %; M = Mean; SD = Standard Deviation; CV = Coefficient of Variation; MD = Mother; FD = Father; CBCL = Child Behaviour Checklist; YSR = Youth Self-Report; CDI = Children’s Depression Inventory; MASC = Multidimensional Anxiety Scale for Children.

**Table 3:** Correlations between “length of stay” and CBCL-mother and YSR-patient scores.

CBCL	Mother (*n* = 148)		YSR (*n* = 163)	
	rho	*p*-Value	rho	*p*-Value
Social Competence	−0.208	0.013 **	−0.028	0.722
Anxious/Depressed	0.208	0.011 **	0.225	0.004 **
Withdrawn/Depressed	0.275	<0.001 ***	0.201	0.011 *
Somatic Complaints	0.213	0.009 **	0.137	0.081
Social Problems	0.109	0.185	0.271	<0.001 **
Thought Problems	0.164	0.046 *	0.166	0.034 *
Attention Problems	0.092	0.267	0.159	0.043 *
Internalizing Problems	0.294	<0.001 ***	0.207	0.008 **
Total Problems	0.165	0.046 *	0.240	0.002 **
Affective Problems	0.288	<0.001 ***	0.245	0.002 **
Anxiety Problems	0.124	0.134	0.247	0.002 **
Obsessive Compulsive Problems	0.225	0.006 **	0.236	0.003 **
Post-traumatic Stress Problems	0.280	<0.001 ***	0.219	0.005 **
Sluggish Cognitive Tempo	0.243	0.003 **		/

Note: * *p* < 0.05, ** *p* < 0.01, *** *p* < 0.001. *n* = Sample size; CBCL = Child Behaviour Checklist; YSR = Youth Self-Report;/= The Sluggish Cognitive Tempo do not exist in currently YSR version.

**Table 4:** Correlations between “length of stay” and CDI and MASC scores.

**CDI (*n* = 68)**	
CDI Total	rho = 0.413, *p* =< 0.001 ***
Negative Mood	rho = 0.282, *p* = 0.020 *
Negative Self-esteem	rho = 0.330, *p* = 0.006 *
**MASC (*n* = 128)**	
Social Anxiety	rho = 0.238, *p* = 0.007 **
Anxiety Disorder Index(ADI)	rho = 0.229, *p* = 0.009 **

Note: * *p* < 0.05, **p < 0.01, *** *p* < 0.001. *n* = Sample size; CDI = Children’s Depression Inventory; MASC = Multidimensional Anxiety Scale for Children.

**Table 5:** Total percentage frequency (%) of sex for cause of admission and discharge diagnosis.

Cause of Admission	Sex	
	F	M
Suicidality	29.6%	10.2%
Non-suicidal self-injury	10.9%	2.3%
Psychomotor agitation/aggressive behavior	10%	36.4%
Eating difficulties and mismanagement	16.1%	8%
Anxiety symptoms	9.1%	4.5%
Psychotic symptoms	3.9%	3.4%
Functional symptoms	8.7%	10.2%
Other	11.7%	25%
**Main Diagnosis (ICD-10)**		
Mood (affective) disorders (F30–F39)	44.8%	27.3%
Behavioral disorders (F90–F98)	7.8%	23.9%
Eating disorders (F50)	12.6%	4.5%
Anxiety disorders (F40–F42)	18.7%	14.8%
Psychotic disorders (F20–F29)	3.9%	5.7%
Somatoform and conversion dis. (F44–F45)	5.7%	6.8%
Dis. of psychological development (F80–F89)	2.6%	4.5%
Other	1.7%	9.1%
No diagnosis	2.2%	3.4%

**Table 6:** Logistic regression.

							95%Confidence Interval	
Predictor		B	SE	Z	*p*	Oddsratio	Lower	Upper
**Intercept**		−22.659	0.7476	−3.031	0.002	0.104	0.0240	0.449
**Age**		0.1221	0.0518	2.355	0.019 *	1.130	10.207	1.251
**FHPD**								
PRESENT–ABSENT		0.8704	0.2845	3.059	0.002 **	2.388	13.672	4.170
**Intrafamily conflict**								
PRESENT–ABSENT		0.0321	0.2864	0.112	0.911	1.033	0.5891	1.810
**Cause of admission:**								
Suicidality–Other		0.3539	0.4620	0.766	0.444	1.425	0.5760	3.524
Anxiety symptoms–Other		0.8722	0.5708	1.528	0.127	2.392	0.7815	7.323
Eating difficulties and mismanagement–Other		18.916	0.5292	3.575	<0.001 ***	6.630	23.502	18.705
Psychomotor agitation/aggressive behavior–Other		10.065	0.4722	2.131	0.033 *	2.736	10.843	6.904
Psychotic symptoms–Other		25.580	11.238	2.276	0.023*	12.910	14.267	116.825
Functional symptoms–Other		0.9034	0.5260	1.717	0.086	2.468	0.8802	6.919
Non-suicidal self-injury–Other		11.836	0.6040	1.960	0.050 *	3.266	0.9997	10.670
**Hospital admission:**								
From ambulatory–Emergency Department		0.3558	0.6403	0.556	0.578	1.427	0.4069	5.007
Scheduled–Emergency Department		−0.9797	0.4148	−2.362	0.018 *	0.375	0.1665	0.846
From transfer or consultancy–Emergency Department		−0.2852	0.3285	−0.868	0.385	0.752	0.3949	1.431
**Comorbidity:**								
Yes–No		0.3018	0.2693	1.121	0.262	1.352	0.7978	2.292

*Note.* Estimates represent the log odds of “Length of Hospitalization = long” vs. “ Length of Hospitalization = brief”; Note: * *p* < 0.05, ** *p* < 0.01, *** *p* < 0.001. Family history of psychiatric disorders (FHPD).

## Data Availability

Datasets analyzed or generated during the study can be requested from the authors.
